# *catena*-Poly[[dinitratocopper(II)]-μ-4,4′′-bis­(1*H*-benzimidazol-1-yl)-1,1′:4′,1′′-terphen­yl]

**DOI:** 10.1107/S1600536811013596

**Published:** 2011-04-16

**Authors:** Hong-Shi Jiang, Hui Li, Jian Wang, Hui-Xuan Ma, Zhe Zhang

**Affiliations:** a Department of Applied Chemistry, Yuncheng University, Yuncheng, Shanxi 044000, People’s Republic of China

## Abstract

In the title one-dimensional coordination polymer, [Cu(NO_3_)_2_(C_32_H_22_N_4_)]_*n*_, the Cu^2+^ ion (site symmetry 2) is coordinated by two nitrate O atoms and two N atoms from two 4,4′-bis­(benzoimidazol-1-yl)terphenyl (*L*) ligands in a distorted *cis*-CuN_2_O_2_ square-planar coordination geometry. An alternative description of the metal coordination geometry, if long Cu—O contacts to the bonded nitrate anions are considered, is an extremely distorted *cis*-CuN_2_O_4_ octa­hedron. The complete *L* ligand is generated by crystallographic twofold symmetry and connects the metal ions into infinite chains propagating in [10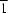]. The dihedral angle between the benzimidazole ring system and the adjacent benzene (*B*) ring is 51.12 (11)° and the dihedral angle between the *B* ring and the central ring is 19.45 (13)°.

## Related literature

For background to benzimidazole-derived ligands in coordin­ation polymers, see: Jin *et al.* (2006 ▶); Li *et al.* (2010 ▶); Su *et al.* (2003 ▶).
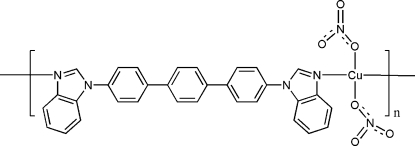


## Experimental

### 

#### Crystal data

[Cu(NO_3_)_2_(C_32_H_22_N_4_)]
*M* *_r_* = 650.10
Monoclinic, 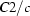

*a* = 14.960 (3) Å
*b* = 15.237 (3) Å
*c* = 12.139 (2) Å
β = 103.94 (3)°
*V* = 2685.7 (9) Å^3^
*Z* = 4
Mo *K*α radiation
μ = 0.88 mm^−1^
*T* = 293 K
0.20 × 0.18 × 0.15 mm

#### Data collection

Rigaku Mercury CCD diffractometer
Absorption correction: multi-scan (*CrystalClear*; Rigaku/MSC, 2005 ▶) *T*_min_ = 0.839, *T*_max_ = 0.877
13493 measured reflections
2371 independent reflections
2181 reflections with *I* > 2σ(*I*)
*R*_int_ = 0.044

#### Refinement

*R*[*F* ^2^ > 2σ(*F* ^2^)] = 0.044
*wR*(*F* ^2^) = 0.124
*S* = 1.09
2371 reflections
204 parameters
H-atom parameters constrained
Δρ_max_ = 0.37 e Å^−3^
Δρ_min_ = −0.77 e Å^−3^

### 

Data collection: *CrystalClear* (Rigaku/MSC, 2005 ▶); cell refinement: *CrystalClear*; data reduction: *CrystalClear*; program(s) used to solve structure: *SHELXS97* (Sheldrick, 2008 ▶); program(s) used to refine structure: *SHELXL97* (Sheldrick, 2008 ▶); molecular graphics: *SHELXTL* (Sheldrick, 2008 ▶); software used to prepare material for publication: *SHELXTL*.

## Supplementary Material

Crystal structure: contains datablocks I, global. DOI: 10.1107/S1600536811013596/hb5843sup1.cif

Structure factors: contains datablocks I. DOI: 10.1107/S1600536811013596/hb5843Isup2.hkl

Additional supplementary materials:  crystallographic information; 3D view; checkCIF report

## Figures and Tables

**Table 1:** Selected bond lengths (Å)

Cu1—N1	1.985 (2)
Cu1—O1	2.025 (2)
Cu1—O3	2.452 (3)

**Fig. 1.**
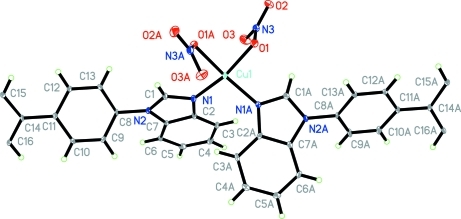
The molecular structure of (I). Displacement ellipsoids are drawn at the 30% probability level and H atoms are shown as small spheres of arbitrary radius.

**Fig. 2.**
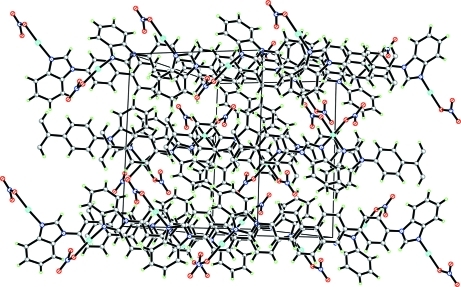
The crystal packing for (I).

## supplementary crystallographic information

### Comment

Benzimidazole has been well used in crystal engineering, and a large number of benzimidazole-containing flexible ligands have been extensively studied (Su *et al.*,2003; Jin *et al.*,2006). However, to our knowledge, the research on benzoimidazole ligands bearing rigid spacers is still less developed (Li *et al.*,2010).

Single-crystal X-ray diffraction analysis reveals that the title compound (I) crystallizes in the monoclinic space group *C2/c*. The geometry of the Cu(II) ion is surrounded by two benzoiimidazole rings of individual L ligands and two nitrate anions, which illustrates a distorted square coordination environment (Fig. 1). Notably, as shown in Fig. 2, the four-coordinated Cu(II) center is bridged by the linear ligand L to form an infinite one-dimensional architecture.

### Experimental

A mixture of CH_3_OH and CHCl_3_ (1:1, 8 ml), as a buffer layer, was carefully layered over a solution of 4,4'-Bis(benzoimidazol-1-yl)terphenyl (L, 0.06 mmol) in CHCl_3_ (6 ml). Then, a solution of Cu(NO_3_)_2_ (0.02 mmol) in CH_3_OH (6 ml) was layered over the buffer layer, and the resultant reaction was left to stand at room temperature. After *ca* three weeks, green block single crystals appeared at the boundary. Yield: ~30% (based on L).

### Refinement

C-bound H atoms were positioned geometrically and refined in the riding-model approximation, with C—H = 0.93Å and *U*_iso_(H) = 1.2Ueq.

### Crystal data

**Table:**

[Cu(NO_3_)_2_(C_32_H_22_N_4_)]	*F*(000) = 1332
*M**_r_* = 650.10	*D*_x_ = 1.608 Mg m^−^^3^
Monoclinic, *C*2/*c*	Mo *K*α radiation, λ = 0.71073 Å
Hall symbol: -C 2yc	Cell parameters from 4661 reflections
*a* = 14.960 (3) Å	θ = 1.9–28.7°
*b* = 15.237 (3) Å	µ = 0.88 mm^−^^1^
*c* = 12.139 (2) Å	*T* = 293 K
β = 103.94 (3)°	Block, green
*V* = 2685.7 (9) Å^3^	0.20 × 0.18 × 0.15 mm
*Z* = 4	

### Data collection

**Table:**

Rigaku Mercury CCD diffractometer	2371 independent reflections
Radiation source: fine-focus sealed tube	2181 reflections with *I* > 2σ(*I*)
graphite	*R*_int_ = 0.044
Detector resolution: 9 pixels mm^-1^	θ_max_ = 25.0°, θ_min_ = 1.9°
ω scans	*h* = −17→17
Absorption correction: multi-scan (*CrystalClear*; Rigaku/MSC, 2005)	*k* = −18→18
*T*_min_ = 0.839, *T*_max_ = 0.877	*l* = −14→14
13493 measured reflections	

### Refinement

**Table:**

Refinement on *F*^2^	Primary atom site location: structure-invariant direct methods
Least-squares matrix: full	Secondary atom site location: difference Fourier map
*R*[*F*^2^ > 2σ(*F*^2^)] = 0.044	Hydrogen site location: inferred from neighbouring sites
*wR*(*F*^2^) = 0.124	H-atom parameters constrained
*S* = 1.09	*w* = 1/[σ^2^(*F*_o_^2^) + (0.0735*P*)^2^ + 6.1716*P*] where *P* = (*F*_o_^2^ + 2*F*_c_^2^)/3
2371 reflections	(Δ/σ)_max_ = 0.001
204 parameters	Δρ_max_ = 0.37 e Å^−^^3^
0 restraints	Δρ_min_ = −0.77 e Å^−^^3^

### Special details

**Table:**

Geometry. All e.s.d.'s (except the e.s.d. in the dihedral angle between two l.s. planes) are estimated using the full covariance matrix. The cell e.s.d.'s are taken into account individually in the estimation of e.s.d.'s in distances, angles and torsion angles; correlations between e.s.d.'s in cell parameters are only used when they are defined by crystal symmetry. An approximate (isotropic) treatment of cell e.s.d.'s is used for estimating e.s.d.'s involving l.s. planes.
Refinement. Refinement of *F*^2^ against ALL reflections. The weighted *R*-factor *wR* and goodness of fit *S* are based on *F*^2^, conventional *R*-factors *R* are based on *F*, with *F* set to zero for negative *F*^2^. The threshold expression of *F*^2^ > σ(*F*^2^) is used only for calculating *R*-factors(gt) *etc*. and is not relevant to the choice of reflections for refinement. *R*-factors based on *F*^2^ are statistically about twice as large as those based on *F*, and *R*- factors based on ALL data will be even larger.

### Fractional atomic coordinates and isotropic or equivalent isotropic displacement parameters (Å^2^)

**Table:**

	*x*	*y*	*z*	*U*_iso_*/*U*_eq_	
Cu1	1.0000	1.08431 (3)	0.7500	0.0158 (2)	
N1	0.90976 (16)	1.00076 (15)	0.7873 (2)	0.0147 (5)	
N2	0.79830 (16)	0.95918 (15)	0.8684 (2)	0.0127 (5)	
N3	1.00584 (19)	1.19959 (17)	0.5927 (2)	0.0228 (6)	
C1	0.86254 (19)	1.01906 (19)	0.8646 (2)	0.0149 (6)	
H1A	0.8733	1.0684	0.9110	0.018*	
C2	0.87224 (19)	0.92203 (18)	0.7368 (2)	0.0124 (6)	
C3	0.89502 (19)	0.87094 (19)	0.6515 (2)	0.0144 (6)	
H3A	0.9423	0.8872	0.6180	0.017*	
C4	0.8443 (2)	0.79544 (19)	0.6192 (2)	0.0176 (6)	
H4A	0.8587	0.7594	0.5641	0.021*	
C5	0.7707 (2)	0.77168 (19)	0.6684 (3)	0.0177 (6)	
H5A	0.7365	0.7217	0.6423	0.021*	
C6	0.74853 (18)	0.82031 (18)	0.7533 (2)	0.0135 (6)	
H6A	0.7009	0.8041	0.7862	0.016*	
C7	0.80152 (19)	0.89544 (18)	0.7875 (2)	0.0119 (6)	
C8	0.73918 (18)	0.96045 (18)	0.9471 (2)	0.0114 (6)	
C9	0.7327 (2)	0.8866 (2)	1.0116 (3)	0.0189 (7)	
H9A	0.7667	0.8365	1.0055	0.023*	
C10	0.6748 (2)	0.88846 (19)	1.0852 (3)	0.0180 (6)	
H10A	0.6696	0.8386	1.1273	0.022*	
C11	0.62384 (18)	0.96369 (18)	1.0978 (2)	0.0113 (6)	
C12	0.63268 (18)	1.03720 (18)	1.0321 (2)	0.0112 (6)	
H12A	0.6000	1.0880	1.0391	0.013*	
C13	0.68927 (19)	1.03586 (18)	0.9565 (2)	0.0116 (6)	
H13A	0.6937	1.0850	0.9127	0.014*	
C14	0.56072 (18)	0.96408 (18)	1.1761 (2)	0.0108 (6)	
C15	0.52982 (19)	1.04264 (18)	1.2139 (2)	0.0129 (6)	
H15A	0.5494	1.0958	1.1903	0.015*	
C16	0.52938 (19)	0.88512 (19)	1.2128 (2)	0.0126 (6)	
H16A	0.5478	0.8320	1.1875	0.015*	
O1	1.06322 (14)	1.18409 (14)	0.68862 (19)	0.0224 (5)	
O2	1.01639 (19)	1.26531 (16)	0.5384 (2)	0.0373 (6)	
O3	0.94347 (17)	1.14482 (17)	0.5587 (2)	0.0352 (6)	

### Atomic displacement parameters (Å^2^)

**Table:**

	*U*^11^	*U*^22^	*U*^33^	*U*^12^	*U*^13^	*U*^23^
Cu1	0.0152 (3)	0.0149 (3)	0.0202 (3)	0.000	0.0099 (2)	0.000
N1	0.0161 (12)	0.0147 (12)	0.0160 (12)	−0.0024 (10)	0.0092 (10)	−0.0034 (10)
N2	0.0140 (12)	0.0135 (12)	0.0132 (12)	0.0014 (9)	0.0084 (10)	−0.0008 (9)
N3	0.0290 (15)	0.0182 (14)	0.0267 (15)	0.0031 (11)	0.0172 (13)	0.0032 (12)
C1	0.0163 (14)	0.0151 (14)	0.0151 (14)	−0.0004 (11)	0.0070 (12)	−0.0004 (12)
C2	0.0091 (13)	0.0161 (14)	0.0119 (14)	−0.0013 (10)	0.0024 (11)	0.0011 (11)
C3	0.0115 (13)	0.0217 (15)	0.0117 (14)	0.0037 (11)	0.0061 (11)	0.0003 (12)
C4	0.0203 (15)	0.0170 (15)	0.0152 (15)	0.0047 (12)	0.0037 (12)	−0.0035 (12)
C5	0.0196 (15)	0.0118 (14)	0.0202 (16)	−0.0010 (12)	0.0020 (12)	−0.0021 (12)
C6	0.0097 (13)	0.0134 (13)	0.0182 (15)	0.0005 (11)	0.0050 (12)	0.0044 (11)
C7	0.0130 (13)	0.0130 (13)	0.0108 (14)	0.0031 (11)	0.0053 (11)	0.0014 (11)
C8	0.0100 (13)	0.0145 (14)	0.0127 (14)	−0.0015 (11)	0.0085 (11)	−0.0011 (11)
C9	0.0200 (16)	0.0161 (15)	0.0262 (17)	0.0077 (12)	0.0164 (13)	0.0053 (13)
C10	0.0199 (15)	0.0145 (15)	0.0239 (16)	0.0048 (12)	0.0140 (13)	0.0085 (12)
C11	0.0103 (13)	0.0148 (14)	0.0097 (13)	−0.0005 (11)	0.0040 (11)	0.0004 (11)
C12	0.0112 (13)	0.0119 (14)	0.0113 (13)	0.0003 (10)	0.0044 (11)	−0.0013 (11)
C13	0.0140 (13)	0.0118 (13)	0.0100 (13)	−0.0020 (11)	0.0048 (11)	0.0015 (11)
C14	0.0096 (13)	0.0156 (14)	0.0086 (13)	0.0006 (10)	0.0046 (11)	0.0010 (11)
C15	0.0134 (13)	0.0118 (14)	0.0156 (14)	−0.0011 (11)	0.0078 (12)	0.0019 (11)
C16	0.0142 (13)	0.0130 (14)	0.0129 (14)	0.0015 (11)	0.0080 (12)	−0.0003 (11)
O1	0.0206 (11)	0.0194 (11)	0.0294 (12)	−0.0026 (9)	0.0102 (10)	−0.0009 (9)
O2	0.0480 (16)	0.0246 (13)	0.0474 (16)	0.0038 (11)	0.0273 (13)	0.0133 (12)
O3	0.0337 (14)	0.0330 (14)	0.0357 (14)	−0.0083 (11)	0.0020 (12)	0.0044 (11)

### Geometric parameters (Å, °)

**Table:**

Cu1—N1	1.985 (2)	C5—C6	1.373 (4)
Cu1—N1^i^	1.985 (2)	C5—H5A	0.9300
Cu1—O1^i^	2.025 (2)	C6—C7	1.398 (4)
Cu1—O1	2.025 (2)	C6—H6A	0.9300
Cu1—O3	2.452 (3)	C8—C9	1.388 (4)
Cu1—O3^i^	2.452 (3)	C8—C13	1.390 (4)
N1—C1	1.333 (4)	C9—C10	1.386 (4)
N1—C2	1.401 (4)	C9—H9A	0.9300
N2—C1	1.334 (4)	C10—C11	1.406 (4)
N2—C7	1.390 (4)	C10—H10A	0.9300
N2—C8	1.450 (3)	C11—C12	1.400 (4)
N3—O2	1.230 (3)	C11—C14	1.493 (4)
N3—O3	1.246 (4)	C12—C13	1.390 (4)
N3—O1	1.291 (4)	C12—H12A	0.9300
C1—H1A	0.9300	C13—H13A	0.9300
C2—C3	1.401 (4)	C14—C15	1.400 (4)
C2—C7	1.406 (4)	C14—C16	1.402 (4)
C3—C4	1.382 (4)	C15—C15^ii^	1.394 (5)
C3—H3A	0.9300	C15—H15A	0.9300
C4—C5	1.420 (4)	C16—C16^ii^	1.404 (5)
C4—H4A	0.9300	C16—H16A	0.9300
			
N1—Cu1—N1^i^	100.24 (14)	C7—C6—H6A	121.9
N1—Cu1—O1^i^	89.66 (9)	N2—C7—C6	131.9 (3)
N1^i^—Cu1—O1^i^	165.63 (9)	N2—C7—C2	105.4 (2)
N1—Cu1—O1	165.63 (9)	C6—C7—C2	122.6 (3)
N1^i^—Cu1—O1	89.66 (9)	C9—C8—C13	120.8 (3)
O1^i^—Cu1—O1	82.68 (12)	C9—C8—N2	119.8 (2)
C1—N1—C2	105.2 (2)	C13—C8—N2	119.4 (2)
C1—N1—Cu1	122.08 (19)	C10—C9—C8	119.1 (3)
C2—N1—Cu1	132.22 (19)	C10—C9—H9A	120.5
C1—N2—C7	107.8 (2)	C8—C9—H9A	120.5
C1—N2—C8	124.9 (2)	C9—C10—C11	121.8 (3)
C7—N2—C8	127.2 (2)	C9—C10—H10A	119.1
O2—N3—O3	123.4 (3)	C11—C10—H10A	119.1
O2—N3—O1	119.3 (3)	C12—C11—C10	117.5 (3)
O3—N3—O1	117.3 (2)	C12—C11—C14	121.4 (2)
N1—C1—N2	112.9 (3)	C10—C11—C14	121.0 (2)
N1—C1—H1A	123.5	C13—C12—C11	121.4 (3)
N2—C1—H1A	123.5	C13—C12—H12A	119.3
C3—C2—N1	131.0 (3)	C11—C12—H12A	119.3
C3—C2—C7	120.4 (3)	C8—C13—C12	119.4 (3)
N1—C2—C7	108.6 (2)	C8—C13—H13A	120.3
C4—C3—C2	117.2 (3)	C12—C13—H13A	120.3
C4—C3—H3A	121.4	C15—C14—C16	117.9 (3)
C2—C3—H3A	121.4	C15—C14—C11	121.4 (2)
C3—C4—C5	121.4 (3)	C16—C14—C11	120.7 (2)
C3—C4—H4A	119.3	C15^ii^—C15—C14	121.21 (16)
C5—C4—H4A	119.3	C15^ii^—C15—H15A	119.4
C6—C5—C4	122.0 (3)	C14—C15—H15A	119.4
C6—C5—H5A	119.0	C14—C16—C16^ii^	120.89 (16)
C4—C5—H5A	119.0	C14—C16—H16A	119.6
C5—C6—C7	116.3 (3)	C16^ii^—C16—H16A	119.6
C5—C6—H6A	121.9	N3—O1—Cu1	101.61 (16)
			
N1^i^—Cu1—N1—C1	147.7 (3)	N1—C2—C7—C6	178.1 (2)
O1^i^—Cu1—N1—C1	−21.8 (2)	C1—N2—C8—C9	−128.1 (3)
O1—Cu1—N1—C1	−79.4 (4)	C7—N2—C8—C9	49.3 (4)
N1^i^—Cu1—N1—C2	−41.6 (2)	C1—N2—C8—C13	52.2 (4)
O1^i^—Cu1—N1—C2	148.9 (3)	C7—N2—C8—C13	−130.4 (3)
O1—Cu1—N1—C2	91.3 (4)	C13—C8—C9—C10	0.6 (4)
C2—N1—C1—N2	0.1 (3)	N2—C8—C9—C10	−179.1 (3)
Cu1—N1—C1—N2	173.00 (18)	C8—C9—C10—C11	−1.2 (5)
C7—N2—C1—N1	−0.3 (3)	C9—C10—C11—C12	0.7 (4)
C8—N2—C1—N1	177.6 (2)	C9—C10—C11—C14	179.0 (3)
C1—N1—C2—C3	−179.0 (3)	C10—C11—C12—C13	0.4 (4)
Cu1—N1—C2—C3	9.1 (5)	C14—C11—C12—C13	−178.0 (3)
C1—N1—C2—C7	0.1 (3)	C9—C8—C13—C12	0.4 (4)
Cu1—N1—C2—C7	−171.8 (2)	N2—C8—C13—C12	−179.9 (2)
N1—C2—C3—C4	−179.9 (3)	C11—C12—C13—C8	−0.9 (4)
C7—C2—C3—C4	1.0 (4)	C12—C11—C14—C15	−19.7 (4)
C2—C3—C4—C5	1.5 (4)	C10—C11—C14—C15	161.9 (3)
C3—C4—C5—C6	−2.7 (4)	C12—C11—C14—C16	159.3 (3)
C4—C5—C6—C7	1.0 (4)	C10—C11—C14—C16	−19.0 (4)
C1—N2—C7—C6	−177.8 (3)	C16—C14—C15—C15^ii^	0.4 (5)
C8—N2—C7—C6	4.5 (5)	C11—C14—C15—C15^ii^	179.4 (3)
C1—N2—C7—C2	0.3 (3)	C15—C14—C16—C16^ii^	−1.5 (5)
C8—N2—C7—C2	−177.4 (3)	C11—C14—C16—C16^ii^	179.5 (3)
C5—C6—C7—N2	179.4 (3)	O2—N3—O1—Cu1	170.1 (2)
C5—C6—C7—C2	1.6 (4)	O3—N3—O1—Cu1	−11.7 (3)
C3—C2—C7—N2	179.0 (3)	N1—Cu1—O1—N3	−28.9 (4)
N1—C2—C7—N2	−0.2 (3)	N1^i^—Cu1—O1—N3	105.01 (17)
C3—C2—C7—C6	−2.7 (4)	O1^i^—Cu1—O1—N3	−87.19 (17)

Symmetry codes: (i) −*x*+2, *y*, −*z*+3/2; (ii) −*x*+1, *y*, −*z*+5/2.
